# THE EDUCATION-HEALTH GRADIENT AND PATHWAYS TO MORTALITY

**DOI:** 10.1093/geroni/igz038.1176

**Published:** 2019-11-08

**Authors:** Justin J Kiel, Yenee Soh, Muqi Guo, David Canning

**Affiliations:** Harvard T.H. Chan School of Public Health, Boston, Massachusetts, United States

## Abstract

Large and widening educational gaps in life expectancy exist in the United States, but the mechanisms behind the education-health gradient are not well understood. We aim to study the different pathways of mortality by education group and decompose the life expectancy gap at age 50 into three components: differences in initial health at age 50, differences in transition rates to poor health states, and differences in mortality rates, given the health state. We use 11 waves of the Health and Retirement Survey and model the evolution of mortality, and health states (measured by self-rated health, limitation on any activity of daily living, and ever diagnosed with a range of health conditions). We estimate Markov models of transitions between health states and mortality over two year periods and use these transition matrices to simulate how a cohort’s health changes as they age. We find a 6-year gap in life expectancy between the highest and lowest education groups. Initial health states account for one-third of the gap in life expectancy, primarily from differences in objective health measures. The gap has widened over time, explained by comparatively better health status changes for the higher educated group, compared to the other education groups. The lower education groups have high rates of transition to poor health states, but not differential mortality rates conditional on their health state. These results suggest that educational gaps in life expectancy are due to the onset of ill health at earlier ages, and not different mortality outcomes after disease diagnoses.

